# The impact on clinical outcome of high prevalence of diabetes mellitus in Taiwanese patients with colorectal cancer

**DOI:** 10.1186/1477-7819-10-76

**Published:** 2012-05-03

**Authors:** Ching-Wen Huang, Li-Chu Sun, Ying-Ling Shih, Hsiang-Lin Tsai, Chao-Wen Chen, Yung-Sung Yeh, Cheng-Jen Ma, Che-Jen Huang, Jaw-Yuan Wang

**Affiliations:** 1 Department of Surgery, Kaohsiung Municipal Hsiao-Kang Hospital, Kaohsiung Medical University, Kaohsiung, Taiwan; 2 Division of Gastroenterological and General Surgery, Department of Surgery, Kaohsiung Medical University Hospital, Kaohsiung Medical University, Kaohsiung, Taiwan; 3 Graduate Institute of Medicine, College of Medicine, Kaohsiung Medical University, Kaohsiung, Taiwan; 4 Nutrition Support Team, Kaohsiung Medical University Hospital, Kaohsiung Medical University, Kaohsiung, Taiwan; 5 Department of Nursing, Kaohsiung Medical University Hospital, Kaohsiung Medical University, Kaohsiung, 807, Taiwan; 6 Division of General Surgery Medicine, Department of Surgery, Kaohsiung Medical, University Hospital, Kaohsiung Medical University, Kaohsiung, Taiwan; 7 Program of Bachelor of Health Beauty, School of Medical and Health Sciences, Fooyin University, Kaohsiung, Taiwan; 8 Division of Trauma, Department of Surgery, Kaohsiung Medical University Hospital, Kaohsiung Medical University, Kaohsiung, Taiwan; 9 Department of Surgery, Faculty of Medicine, College of Medicine, Kaohsiung Medical University, Kaohsiung, Taiwan; 10 Cancer Center, Department of Surgery, Kaohsiung Medical University Hospital, Kaohsiung Medical University, Kaohsiung, Taiwan; 11 Department of Medical Genetics, College of Medicine, Kaohsiung Medical University, Kaohsiung, Taiwan; 12 Department of Surgery, Faculty of Medicine, College of Medicine and Kaohsiung Medical University Hospital, Kaohsiung Medical University, 100 Tzyou 1st Road, San-Ming District, Kaohsiung, 807, Taiwan

**Keywords:** High prevalence, Diabetes mellitus, Colorectal cancer, Survival impact

## Abstract

**Background:**

Both colorectal cancer (CRC) and diabetes mellitus (DM) are important public health problems worldwide. As there are controversies about survival impact on CRC patients with preexisting DM, the purpose of the present study is to evaluate the incidence and the survival impact of preexisting DM on the long-term outcomes of patients with CRC in Taiwan.

**Methods:**

From January 2002 to December 2008, 1,197 consecutive patients with histologically proven primary CRC, who received surgical treatment at a single institution, were enrolled. The clinicopathologic features between these patients with and without DM were retrospectively investigated. Moreover, we intended to analyze the impact of DM on overall survival (OS) and cancer-specific survival (CSS) rates.

**Results:**

Of 1,197 CRC patients, 23.6% of patients had either a reported history of DM or were currently taking one or more diabetes-controlling medications. CRC patients with DM were significantly older than those without DM (*P* < 0.001), and had a higher incidence of cardiac disease and higher body mass index than those without DM (both *P* < 0.001). There were no significant differences in gender, tumor size, tumor location, histological type, AJCC/UICC cancer stage, vascular invasion, perineural invasion, comorbidity of pulmonary disease or renal disease, and OS, and CSS between two groups. Additionally, DM patients had a higher incidence of second malignancy than patients without DM (9.54% *vs* 6.01%, *P* = 0.040).

**Conclusions:**

A considerably high prevalence of DM in CRC patients but no significant impact of DM on survival was observed in the single-institution retrospective study, regardless of cancer stages and tumor locations. Therefore, treatment strategies for CRC patients with DM should be the same as patients without DM.

## Background

Colorectal cancer (CRC) is reported as the third most common cancer and the second leading cause of cancer death in the United States [1,2]. In the United States, an estimated 142,570 newly diagnosed cases of CRC and an estimated 51,370 cancer deaths from CRC were reported in 2010 [1]. It has been reported that the incidence of CRC in economically transitioning countries continues to rise and the incidence of CRC in economically developed countries has stabilized or is declining [3,4]. There was a 33.36% decrease CRC-related death rate in 2006 compared with that in 1990 [1]. In Taiwan, CRC is the third leading cause of cancer-related deaths and the death rate was 19.6 per 100,000 in 2009 [5]. Furthermore, there was a 26.45% increase in the CRC-related death rate in 2009 compared with that in 2001 and a 59.35% increase compared with that in 1996 [5].

Diabetes mellitus (DM) is one of the most important public problems worldwide. The International Diabetes Federation estimates that 285 million people around the world have diabetes, and the total patient number is expected to rise to 438 million within 20 years [6]. Because a Western style diet, sedentary lifestyle and obesity are the risk factors of DM, the prevalence and incidence of DM have increased rapidly. The prevalence of DM is 3 to 7% in economically developed countries and 2 to 5% in economically transitioning countries [6]. In Taiwan, the age and gender-adjusted prevalence of DM is 6.6% for the general population; meanwhile, the prevalence of DM is 20.2% in populations older than 60 years [5]. In Taiwan, DM is the fifth leading cause of death and the death rate was 35.66 per 100,000 in 2009 [5]. DM has been shown to be associated with increased risk of many types of cancer, including liver [7], pancreatic [8-10], endometrial [11], colorectal [12-19], breast [20], and bladder [21]. A Western style diet, sedentary lifestyle and obesity are the risk factors for both DM and CRC; consequently, many studies have shown a 24 to 60% increased risk of developing CRC in DM patients [12-19].

Though the impact of preexisting diabetes on the outcomes of patients with newly diagnosed CRC has been evaluated previously, results have varied from different countries. A significantly higher rate of overall mortality and cancer recurrence was found in patients with DM and high-risk stage II and stage III colon cancer [22]. Coughlin *et al*. reported that diabetes was significantly associated with fatal colon cancer in men and women [23]. Similarly, Huang *et al*. showed that diabetes is a poor prognostic factor in patients with newly diagnosed colon cancer, and it may directly impact the tumor behavior of stage II disease [24]. On the contrary, some investigators demonstrated that DM did not affect the short-term survival or the cancer specific survival [25]. There are controversies about the impact of preexisting diabetes on the outcomes of patients with newly diagnosed CRC. Therefore, we conducted a retrospective study to evaluate the survival impact of preexisting diabetes on the outcomes of Taiwanese patients with newly diagnosed CRC.

## Methods

### Patients

This retrospective cohort study included 1,197 consecutive patients with histologically proven CRC, who received surgical treatment with curative intent from a single-institution, Kaohsiung Medical University Hospital, from January 2002 to December 2008. The present study was approved by the Institutional Review Board of the Kaohsiung Medical University Hospital. Patients’ clinical outcomes and survival statuses were regularly followed up. Available variables included: age of onset, sex, tumor location, histological type, TNM classification defined according to the criteria of the American Joint Commission on Cancer (AJCC) [26], vascular invasion, perineural invasion, preoperative serum level of albumin, preoperative and postoperative serum level of CEA, comorbidity of cardiac disease, pulmonary disease, and renal disease, chemotherapy, and body mass index (BMI). The diagnoses of DM were made according to the chart record of a history of DM or taking medicines for DM. Preoperative serum levels of albumin and CEA were checked within one week before the operation, and postoperative serum levels of CEA were checked at least four weeks after. The cut-off values of serum albumin and CEA were set at 3.5 gm/dl and 5 ng/ml, respectively. The diagnosis of diabetes mellitus was made according to the records of the charts. The existences of comorbidity were according to the chart record of International Classification of Diseases (ICD, 9th version), that are ICD codes 390 to 398, 410 to 414, and 420 to 429 for cardiac disease, 490 to 496 for pulmonary disease, 584 to 588 for renal disease.

All patients were followed up until their deaths, or untill December 2010. The median follow-up time was 32 months (range: 1 to 96 months). Cancer-specific survival was defined as the time elapsed between primary surgery and death from CRC. Overall survival was defined as the time elapsed between primary surgery and death from any cause.

### Statistical analysis

All data were statistically analyzed using the Statistical Package for the Social Sciences, version 17.0 (SPSS Inc., Chicago, IL, USA). For the univariate statistical analysis, a Chi-square test was used where applicable. A Cox proportional hazards model with forward stepwise variable selection was used for multivariate testing of those factors found to be significant by univariate analysis (the inclusion factors were those with *P-*value less than 0.05 by univariate analysis). Overall and cancer-specific survival rates (OS and CSS) were calculated by the Kaplan-Meier method, and the differences in survival rates were analyzed by the log-rank test. A *P-*value less than 0.05 was considered to be statistically significant.

## Results

### Characteristics of colorectal cancer patients

The clinical and pathologic data regarding 1,197 CRC patients are summarized in Table 1. There were 283 (23.6%) patients diagnosed with diabetes mellitus. Patients in the DM group were significantly older than patients in the non-diabetes group (67.63 ± 10.55 *vs* 63.11 ± 13.45, *P* <0.001). Low preoperative serum albumin level was prominently encountered in patients with DM when compared to patients without DM (44.9% *vs* 38.2%, *P* =0.018). Higher preoperative and postoperative serum CEA were more frequently observed in patients with DM when compared to patients without DM (56.6% *vs* 43.8%, *P* <0.001; 32.9% *vs* 26.8%, *P* =0.024, respectively). Higher percentage of concurrent cardiac disease was also noted in patients with DM when compared to patients without DM (61.1% *vs* 34.0%, *P* <0.001). Further, BMI was significantly higher in patients with DM than patients without DM (24.36 ± 3.65 *vs* 23.29 ± 3.75, *P* <0.001). However, there were no significant differences in gender, tumor size, tumor location, histological type, AJCC/UICC cancer stage, vascular invasion, perineural invasion, the percentages of patients receiving chemotherapy, and comorbidity of pulmonary disease and renal disease. In addition, DM patients had a higher incidence of second primary malignancy than patients without DM (9.54% *vs* 6.01%, *P* = 0.040).

**Table 1:** Baseline characteristics of 1197 colorectal cancer patients by diabetes mellitus and non- Ddiabetes mellitus status

**Characteristic**	**Diabetes (%)**	**Non-diabetes (%)**	**P value**
	N = 283 (23.6%)	N = 914 (76.4%)	
Age (mean ± SD)	67.63 ± 10.55	63.11 ± 13.45	< 0.001
Gender			
Male/Female	157 (55.5)/126 (45)	516 (56.5)/398 (43.5)	0.772
Tumor size			
≥5 cm/<5 cm	116 (41)/167 (59)	404 (44.2)/510 (55.8)	0.315
Tumor location			
Colon/Rectum	199 (70.3)/84 (29.7)	629 (68.8)/285 (31.2)	0.633
Histological type			
Well/Moderately/Poorly	23 (8.1)/230 (81.3)/30 (10.6)	68(7.4)/747(81.8)/99(10.8)	0.582
AJCC^a^ Stage			
I/II/III/IV	52(18.3)/109(38.5)/85(30)/37(13.2)	155(17)/316(34.6)/280(30.6)/163(17.8)	0.396
Tumor depth			
T1/T2/T3/T4	20(7.0)/43(15.2)/210(74.2)/10(3.6)	61(6.7)/136(14.9)/652(71.3)/67(7.1)	0.305
Lympho Nodes metastases			
N0/N1/N2	171(60.4)/76(26.9)/36(12.7)	523(57.2)/233(25.5)/158(17.3)	0.322
Vascular invasion			
Yes/No	95 (33.6)/188 (66.4)	614 (67.2)/300 (32.8)	0.764
Perineurial invasion			
Yes/No	102 (36)/181 (64)	344 (37.6)/570 (62.4)	0.689
Serum Albumin level			
<3.5 gm/dl/≥3.5 gm/dl	127 (44.9)/156 (55.1)	349 (38.2)/565 (61.8)	0.018
Pre-op Serum CEA^b^ level			
≥5 ng/ml/<5 ng/ml	160 (56.6)/123 (43.5)	400 (43.8)/514 (56.2)	< 0.001
Post-op Serum CEA^b^ level			
≥5 ng/ml/<5 ng/ml	93 (32.9)/190 (67.1)	245 (26.8)/669 (73.2)	0.024
Cardiac disease			
Yes/No	173 (61.1)/110 (38.9)	311 (34)/603 (66)	< 0.001
Pulmonary disease			
Yes/No	4 (1.4)/279 (98.6)	12 (1.3)/902 (98.7)	0.898
Renal disease			
Yes/No	15 (5.3)/268 (94.7)	40 (4.4)/874 (95.6)	0.517
Second Primary Cancer			
Yes/No	27 (9.5)/256 (90.5)	55 (6)/859 (94)	0.040
Chemotherapy			
Yes/No	185 (65.4)/98 (35.6)	607 (66.4)/307 (33.6)	0.756
Body Mass Index (mean ± SD)	24.36 ± 3.65	23.29 ± 3.75	< 0.001

^a^ AJCC American Joint Commission on Cancer.

^b^ CEA Carcinoembryonic antigen.

### Impact on overall survival (OS) and cancer-specific survival (CSS)

The results of prognostic factors on OS for CRC patients are shown in Table 2. Using univariate analysis, we found that older age (*P* = 0.004), tumor location at rectum (*P* = 0.048), larger tumor size (*P* < 0.001), lower BMI (*P* = 0.001), lower pre-operative serum albumin level (*P* < 0.001), poorly differentiated histology (P < 0.001), advanced AJCC/UICC stage (*P* < 0.001), higher pre-operative and post-operative serum CEA levels (both *P* < 0.001), presence of vascular invasion (*P* < 0.001) and perineural invasion (*P* < 0.001) were statistically significant poor prognostic factors of OS. Using multivariate analysis, we found that older age (*P* = 0.021), tumor location at rectum (*P* = 0.048), lower pre-operative serum albumin level (*P* = 0.037), poorly differentiated histology (P = 0.030), advanced AJCC/UICC stage (*P* = 0.037), post-operative serum CEA level (*P* < 0.001), presence of vascular invasion (*P* < 0.001) and perineural invasion (*P* < 0.001) were statistically significant poor prognostic factors of OS. However, DM was not significantly correlated to OS by univariate analysis (*P* = 0.888) and multivariate analysis (*P* = 0.642). The results of prognostic factors on CSS for CRC patients are shown in Table 3. Using univariate analysis, we found that larger tumor size (*P* < 0.001), lower BMI (*P* < 0.001), lower pre-operative serum albumin level (*P* < 0.001), poorly differentiated histology (*P* < 0.001), advanced AJCC/UICC stage (*P* < 0.001), higher pre-operative and post-operative serum CEA levels (both *P* < 0.001), presence of vascular invasion (*P* < 0.001) and perineural invasion (*P* < 0.001) were statistically significant poor prognostic factors of CSS. Using multivariate analysis, we found that lower pre-operative serum albumin level (*P* =0.011), advanced AJCC/UICC stage (*P* < 0.001), higher post-operative serum CEA level (*P* < 0.001), presence of vascular invasion (*P* = .002) and perineural invasion (*P* < 0.001) were statistically significant poor prognostic factors of CSS. However, DM remained not significantly correlated to CSS by univariate analysis (*P* = 0.888) and multivariate analysis (*P* = 0.234).

**Table 2:** Univariate and multivariate analysis of prognostic indicators on overall survival for colorectal cancer patients

**Parameters**	**Number**	**Univariate analysis Hazard ratio (95% CI)**	***P* value**	**Multivariate analysis Hazard ratio (95% CI)**	***P* value**
DM (yes/no)	283/914	1.02(0.81-1.27)	.888	0.94(0.71-1.24)	0.642
Age (≥65/<65)years	632/565	1.33(1.09-1.62)	.004	1.36(1.05-1.76)	0.021
Sex (Male/Female)	674/523	1.13(0.93-1.37)	.221	1.09(0.85-1.40)	0.491
Location (Rectum/Colon)	369/828	0.89(0.65-1.00)	.048	0.80(0.61-1.05)	0.114
Tumor size (≥5/<5)cm	518/679	1.47(1.21-1.78)	< .001	1.12(0.87-1.44)	0.368
BMI^a^ (<22/≥22) kg/M^2^	394/803	1.39(1.13-1.70)	.001	1.14(0.88-1.48)	0.311
Albumin (<3.5/≥3.5)gm/dl	476/721	1.83(1.48-2.25)	< .001	1.32(1.02-1.70)	0.037
Histology (PD/MD + WD^b^)	135/1062	1.93(1.48-2.52)	< .001	1.45(1.04-2.02)	0.030
AJCC^c^ stage (III&IV/I&II)	565/632	3.36(2.73-4.14)	< .001	1.32(1.02-1.70)	0.037
Pre-op CEA^d^ (≥5/<5) ng/ml	559/638	2.86(2.31-3.53)	< .001	1.26(0.93-1.70)	0.143
Post-op CEA^d^ (≥5/<5) ng/ml	338/859	5.30(4.30-6.54)	< .001	3.61(2.80-4.64)	< 0.001
Vascular invasion (yes/no)	395/802	2.79(2.28-3.41)	< .001	1.58(1.22-2.05)	< 0.001
Perineurial invasion (yes/no)	447/750	2.27(1.86-2.78)	< .001	1.79(1.39-2.30)	< 0.001

^a^ BMI Body mass index.

^b^ PD Poorly differentiated, MD Moderately differentiated, WD Well differentiated.

^c^ AJCC American Joint Commission on Cancer.

^d^ CEA Carcinoembryonic antigen.

**Table 3:** Univariate and multivariate analysis of prognostic indicators on cancer-specific survival for colorectal cancer patients

**Parameters**	**Number**	**Univariate analysis Hazard ratio (95% CI)**	***P* value**	**Multivariate analysis Hazard ratio (95% CI)**	***P* value**
DM (yes/no)	283/912	0.91(0.72-1.17)	.463	0.83(0.61-1.23)	0.234
Age (≥65/<65)years	632/565	1.19(0.97-1.46)	.092	1.27(0.97-1.67)	0.083
Sex (Male/Female)	674/523	1.08(0.88-1.32)	.467	1.05(0.81-1.37)	0.692
Location (Rectum/Colon)	369/828	0.81(0.64-1.00)	.058	0.82(0.62-1.09)	0.176
Tumor size (≥5/<5)cm	518/679	1.42(1.15-1.74)	< .001	1.13(0.86-1.47)	0.384
BMI^a^ (<22/≥22) kg/M^2^	394/803	1.43(1.16-1.77)	< .001	1.16(0.88-1.51)	0.301
Albumin (<3.5/≥3.5)gm/dl	476/721	1.79(1.44-2.23)	< .001	1.40(1.08-1.82)	0.011
Histology (PD/MD + WD^b^)	135/1062	1.89(1.42-2.52)	< .001	1.35(.094-1.94)	0.124
AJCC^c^ stage (III&IV/I&II)	565/632	3.95(3.15-4.96)	< .001	2.22(1.65-2.99)	< 0.001
Pre-op CEA^d^ (≥5/<5) ng/ml	559/638	2.98(2.38-3.73)	< .001	1.25(0.91-1.73)	0.225
Post-op CEA^d^ (≥5/<5) ng/ml	338/859	5.47(4.39-6.81)	< .001	3.71(2.84-4.83)	< 0.001
Vascular invasion (yes/no)	395/802	2.97(2.40-3.67)	< .001	1.54(1.18-2.01)	0.002
Perineurial invasion (yes/no)	447/750	2.51(2.03-3.10)	< .001	1.82(1.40-2.37)	< 0.001

^a^ BMI Body mass index.

^b^ PD Poorly differentiated, MD Moderately differentiated, WD Well differentiated.

^c^ AJCC American Joint Commission on Cancer.

^d^ CEA Carcinoembryonic antigen.

### Survival impact of DM in colorectal cancer patients

**Figure 1:**
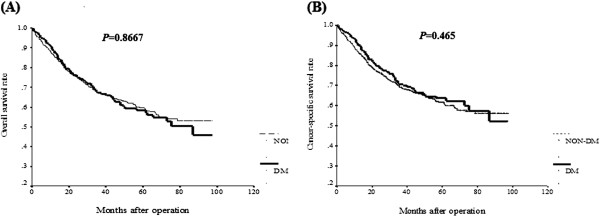
Overall survival (A) and cancer-specific survival (B) for CRC patients by diabetes status.

**Figure 2:**
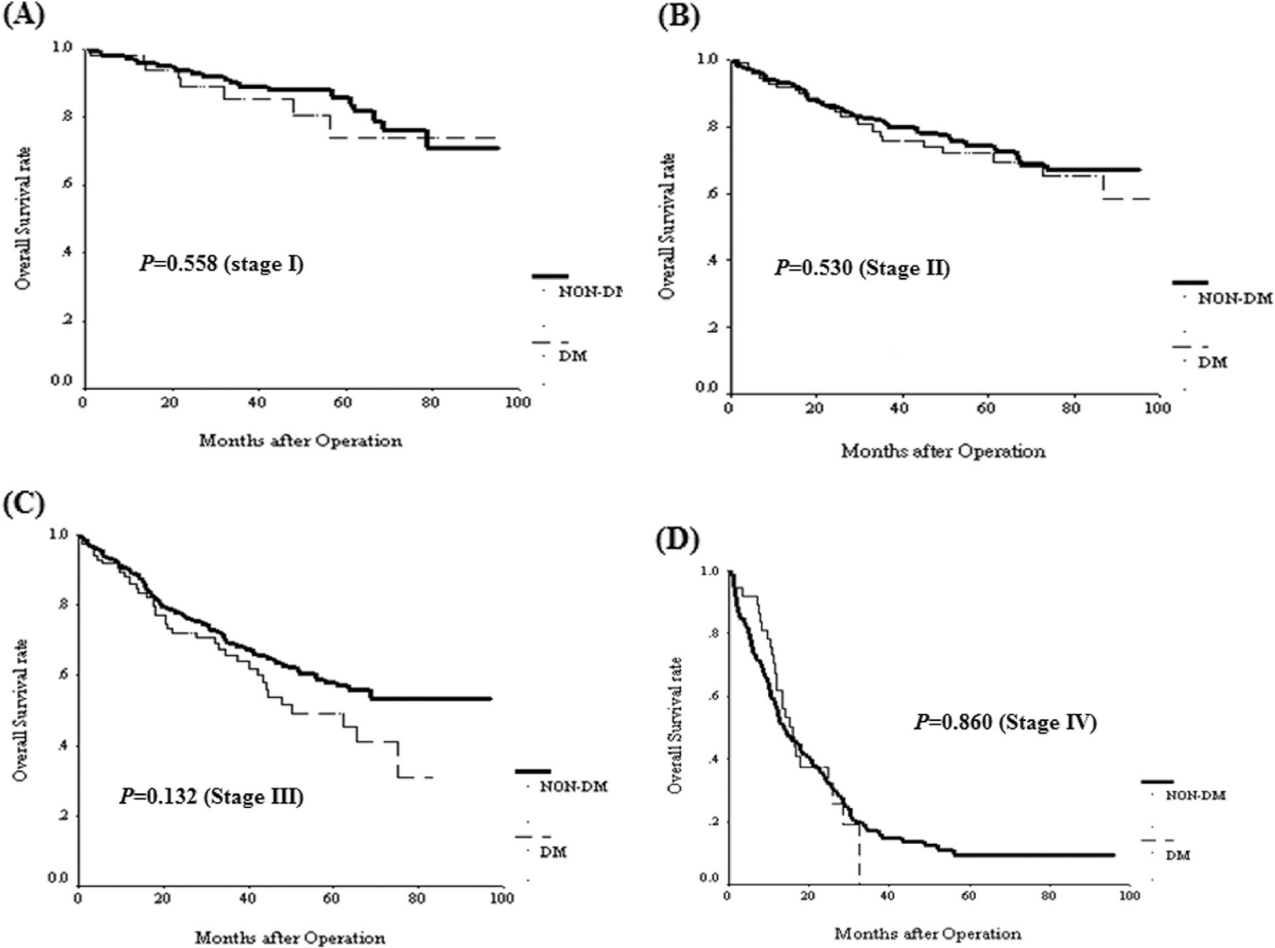
Overall survival for CRC patients by stage status between diabetes and non-diabetes status.

**Figure 3:**
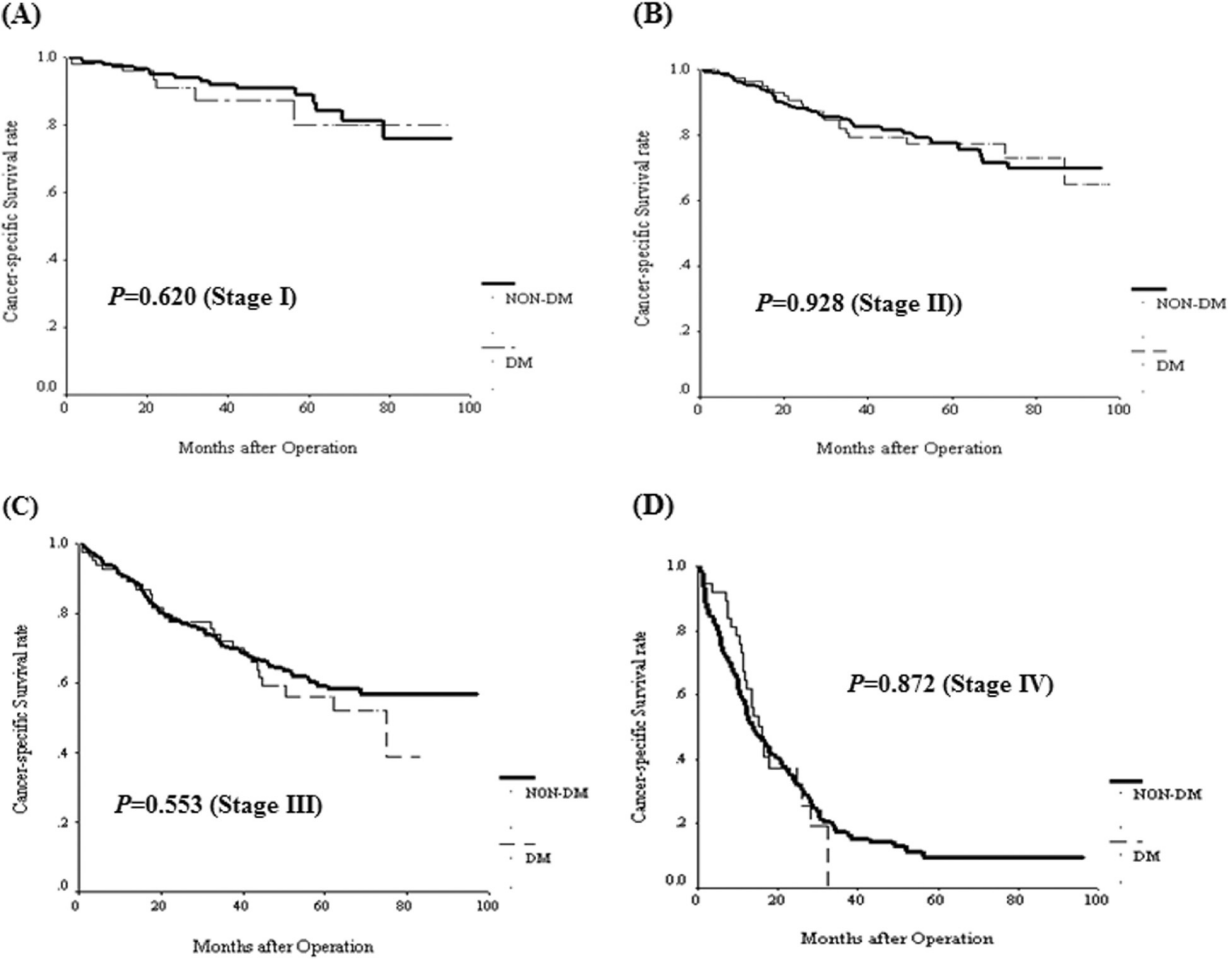
Cancer-specific survival for CRC patients by stage status between diabetes and non-diabetes status.

The Kaplan Meier survival analysis showed that both OS (Figure 1A) and CSS (Figure 1B) were not significantly different between the two groups. Furthermore, we analyzed the impact of DM on OS (Figure 2) and CSS (Figure 3) according to various cancer stages. No statistical differences were found in each cancer stage in either OS or CSS between the two groups. Furthermore, we analyzed the impact of DM on OS and CSS according to tumor location. OS measures were not significantly different between either colon or rectal cancer patients with and without DM (Additional file 1: Figure S1). Likewise, CSS measures were also not significantly different between either colon or rectal cancer patients with and without DM (Additional file 2: Figure S2).

## Discussion

In the present study, we found a considerably higher prevalence of DM in Taiwan CRC patients (23.6%) than previous reports. However, there was no significant survival impact of DM on survival (OS and CSS) in these patients. DM has been reported previously to be associated with increased risk of CRC [12-19]. In our 1197 CRC patients, 283 patients (23.6%) had preexisting DM. Barone *et al*. conducted two meta-analysis systematic reviews and found 8 to 18% of CRC patients with preexisting DM [27,28]. Similarly, Stein *et al*. reported 2 to 18% of comorbidity of CRC and DM in a meta-analysis systematic review [29]. The prevalence of DM in CRC patients varied between 2.8% and 14% in recent other studies from various countries [22,25,30-33]. Our recent study and another study from Huang *et al*. [24] showed that the coexistence of DM and CRC in Taiwanese patients were relatively higher (17% and 23.6%, respectively) than data from other studies.

In the current study, the patients in the DM group were significantly older than the patients in the non-diabetes group. Consistent with our study, two recent studies also indicated that patients with diabetes were significantly older than those without diabetes, and no significant difference was found with regard to gender, tumor stage, or histological grade [24,25]. Chen *et al*. noted that diabetic patients were on average 5.3 years older than non-diabetic patients, but it was not statistically significant [33], whereas Chiao *et al*. reported that age was not significantly different between those with diabetes compared to non-diabetics [34]. They noted that patients with diabetes had significantly higher BMI. Regarding prognostic factors, age was significantly correlated to OS, but not significantly correlated to CSS. Our study team also demonstrated previously that older age may be associated with cardiovascular diseases or other medical illness, and with the significantly higher American Society of Anesthesiologists (ASA) classifications; hence, older age is associated with poor OS, but not CSS in CRC patients [35]. Serum albumin level, histology, AJCC/UICC stage, post-operative serum CEA levels, vascular invasion, and perineural invasion were previously reported as prognostic factors of CRC [35-40], and we also identified these factors were significantly correlated to OS and CSS in the current investigation.

In fact, our findings suggested no survival impact of preexisting DM on OS, and the overall mortality rates between patients with DM and those without DM were similar (35.3% *vs* 35.1%). Our findings were consistent with other studies [33,34,41,42]. However, there are controversies about survival impact of preexisting DM on OS. It was reported that patients with DM, compared with patients without DM, experienced a significantly worse five-year OS [22,24,25,29,31,43]. Again, our findings also suggested no survival impact of preexisting DM on CSS, in consistency with other studies [15,31]. In contrast, there were studies reporting that diabetes confers increased risk for long-term cancer-specific mortality [29,43]. There are still controversies about survival impact of preexisting DM on CSS. Coughlin *et al*. suggested that diabetes was significantly associated with fatal colon cancer in men and women; however, diabetes was not significantly associated with fatal rectal cancer in men and women [23]. Regarding CSS according to tumor location, CSS was not significantly different between colon cancer patients with DM and those without DM, and also not significantly different between rectal cancer patients with DM and those without DM in our studies. Huang *et al*. identified DM as a poor prognostic factor for CSS, particularly in patients with stage II colon cancer [24].

The decision-making of clinical treatment for CRC will be probably influenced by the preexisting DM. van de Poll-Franse *et al*. noted that colon cancer and rectal cancer patients with diabetes were more likely to receive surgery and less likely to receive chemotherapy. Meanwhile, rectal cancer patients with diabetes were less likely to receive (adjuvant) radiotherapy [44]. They suggested that this might be due to concurrent cardiovascular diseases or worse functional status. In the present study and another study [25], higher percentages of concurrence of cardiac disease were noted in patient with DM when compared to patients without DM. Chiao *et al*. also reported patients with diabetes were less likely to receive chemotherapy and radiotherapy compared to those without diabetes [34]. However, some studies reported no significant difference for cancer treatment (chemotherapy and surgery) [24,25,30,33]. Huang *et al*. pointed out that there was a 5.2% absolute difference in the proportion of complete adjuvant in stage II patients; despite this difference, statistical significance was not achieved [24]. In the present study, the percentages of patients receiving chemotherapy were not significantly different in the two groups. Consequently, the similar survival outcomes (OS and CSS) may partially result from the similar treatment modalities between the two groups.

There are some limitations of the present study. First, the present study is a single-institution retrospective study. The diagnosis of DM was made according to the records of the charts. Therefore, some patients who had DM may be diagnosed to be non-DM. Second, the severities of comobidities were not assessed. The presence of a comorbidity occurs in only a relatively small group of patients, and is also the limitation of the current study for the further stratification of severities of comorbidity between DN and non-DM patients. Third, the types of treatment, length of treatment, and, treatment-related toxicities were also not assessed. Consequently, the present study is not necessarily representative of Taiwan as a whole. Finally, as in the majority of the literature, we did not differentiate the types of DM. The mean age of the patients with diabetes was 67 years; the majority of patients with diabetes were supposed to have type 2 DM. In addition, the unavailability of serum level of HbA1c is also a limitation of the present study. Siddiqui *et al*. reported that elevated HbA1c is an independent predictor of aggressive clinical behavior in patients with CRC [42]. In patients with type 2 DM who have CRC, poor glycemic control is associated with a clinically aggressive course for the cancer. The mean values of HbA1c were significantly improved within the controlled range in the year after CRC diagnosis [34]; hence, the quality of DM care before and after CRC diagnosis positively impacted survival among diabetics. Because not all the HbA1c data are available, we cannot demonstrate whether no significant survival impact of DM on survival is due to good quality of DM care. However, because the accessibility of DM care, including clinic visits and medicine administrations, is very convenient and the medical fee is largely covered by the Taiwan National Health Bureau Program, the qualities of DM care are supposed to be favorable.

## Conclusions

Our findings suggest that a prominently high prevalence of DM (23.6%) is a crucial issue of public health in our CRC patients. There is no significant survival impact of DM on survival (OS and CSS) in patients with newly diagnosed CRC; therefore, CRC patients with DM should be treated with the same modalities (including surgery and chemotherapy) as those without DM. More large-scaled multiple center studies are needed to ascertain the impact of DM on the clinical outcome of CRC patients.

## Abbreviations

AJCC: American Joint Commission on Cancer; BMI: Body mass index; CEA: Carcinoembryonic antigen; CRC: Colorectal cancer; CSS: Cancer-specific survival; DM: Diabetes mellitus; MD: Moderately differentiated; OS: Overall survival; PD: Poorly differentiated; WD: Well differentiated.

## Competing interests

The authors declare that they have no competing interest.

## Authors’ contributions

CWH and LCS analyzed the data and wrote the manuscript. YLS, HLT, CWC, YSY, CJM, and CJH made substantial contributions to data acquisition, statistical analyses and data interpretation, and helped in manuscript preparation. JYW participated in study design and coordination. All authors read and approved the final manuscript.

## Supplementary Material

Additional file 1 **Figure S1**. Overall survival for CRC patients by tumor location between diabetes and non-diabetes status. Click here for file

Additional file 2 **Figure S2**. Cancer-specific survival for CRC patients by tumor location between diabetes and non-diabetes status. Click here for file
