# Supplementary figures and images for: The impact on clinical outcome of high prevalence of diabetes mellitus in Taiwanese patients with colorectal cancer
Source: World J Surg Oncol. 2012 May 3;10:76. doi: 10.1186/1477-7819-10-76 (PMC3533895; doi:10.1186/1477-7819-10-76)

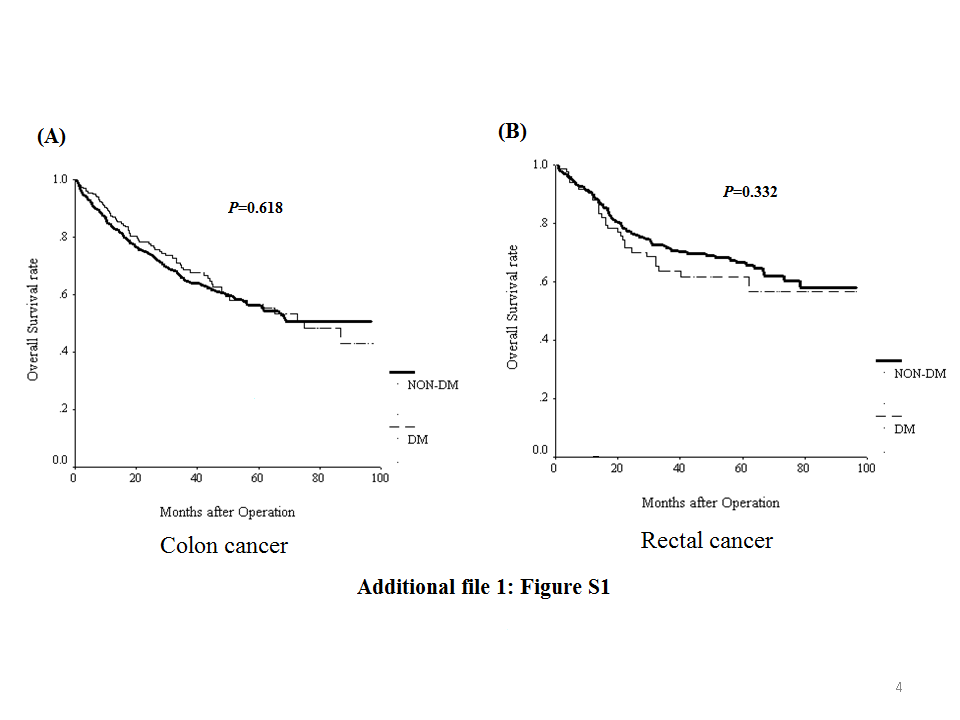

Supplement: Additional file 1 — Figure S1. Overall survival for CRC patients by tumor location between diabetes and non-diabetes status. [file 1477-7819-10-76-S1.tiff]

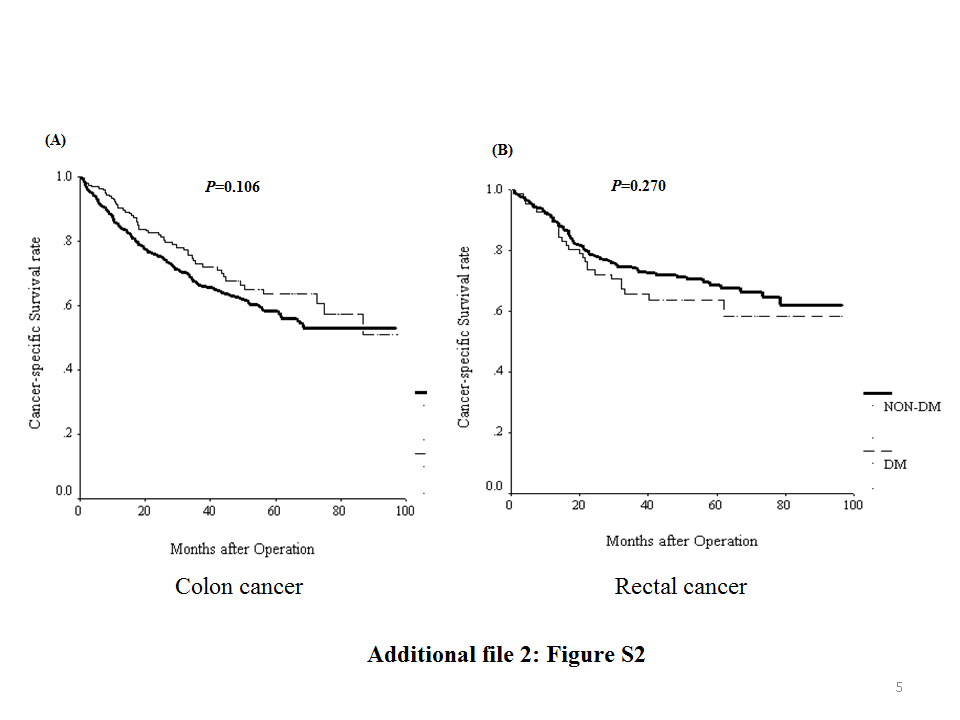

Supplement: Additional file 2 — Figure S2. Cancer-specific survival for CRC patients by tumor location between diabetes and non-diabetes status. [file 1477-7819-10-76-S2.tiff]
